# Health Care Expenditure and GDP in Oil Exporting Countries: Evidence from OPEC Data, 1995-2012

**DOI:** 10.5539/gjhs.v8n2p93

**Published:** 2015-06-11

**Authors:** Ali Akbar Fazaeli, Hossein Ghaderi, Masoud Salehi, Ali Reza Fazaeli

**Affiliations:** 1 Departments of Health Economics, School of Health Management and Information Sciences, Iran University of Medical Sciences, Tehran, IR Iran; 2 Department of Biostatistics, Faculty of Public Health, Iran University of Medical Sciences, Tehran, Iran; 3 Health Management and Economics Research Center, Iran University of Medical Sciences, Tehran, Iran; 4 Fellowship of Rhomatology, Rasoul Akram General Hospital, Iran University of Medical Sciences, Tehran, Iran

**Keywords:** health expenditures, panel data cointegration, GDP, OPEC, luxury good

## Abstract

**Background:**

There is a large body of literature examining income in relation to health expenditures. The share of expenditures in health sector from GDP in developed countries is often larger than in non-developed countries, suggesting that as the level of economic growth increases, health spending increase, too.

**Objectives:**

This paper estimates long-run relationships between health expenditures and GDP based on panel data of a sample of 12 countries of the Organization of the Petroleum Exporting Countries (OPEC), using data for the period 1995-2012.

**Patients & Methods:**

We use panel data unit root tests, cointegration analysis and ECM model to find long-run and short-run relation. This study examines whether health is a luxury or a necessity for OPEC countries within a unit root and cointegration framework.

**Results:**

Panel data analysis indicates that health expenditures and GDP are co-integrated and have Engle and Granger causality. In addition, in oil countries that have oil export income, the share of government expenditures in the health sector is often greater than in private health expenditures similar developed countries.

**Conclusions:**

The findings verify that health care is not a luxury good and income has a robust relationship to health expenditures in OPEC countries.

## 1. Introduction

One of the most main subjects in health is what determines the funds a country devotes to health. The share of health spending in GDP in developed countries is often larger than in developing countries; so, as the level of development decreases, health expenditures decrease, too (Baltagi & Moscone, 2010). A study by Newhouse identified income as the most important factor accounting for differences across countries in the level of health expenditures (Newhouse, 1977). He inferred from a regression of health expenditures per capita on GDP in an OECD report that health is a luxury good. In the other words, health care has high income elasticity of demand in OECD. As countries become wealthier, they purchase more health care. Early studies used a one year to get cross-country estimates of the relationship between health and GDP. These researchers showed income elasticity higher than unity, implying that the share of health expenditures in GDP will increase with the per capita GDP. For some years, economists have discussed whether the income elasticity of health shows that health is a luxury good (Newhouse, 1977); recent studies have used panel data that calculated across countries and time. Using panel increases the sample size and newer studies have used this technique to test and estimate the effect of GDP on health expenditures based on unit root test and cointegration analysis. (Parkin, McGuire, & Yule, 1987) showed that health care is a luxury, (Hansen & King, 1996) indicated a strong relationship between health care and GDP in OECD countries; their results showed that if elasticity of health care is above one, then health care is a luxury. (McCoskey & Selden, 1998) presented unit root test for time series health expenditures and GDP in the OECD. (Anderson, Hurst, Hussey, & Jee-Hughes, 2000) suggested that additional health care spending may indeed generate benefits in OECD. (Devlin & Hansen, 2001) examined the causality between health expenditures and GDP, whereas (Gerdtham & Löthgren, 2002) indicated that health care expenditures and GDP are cointegrated. (Di Matteo, 2003) demonstrated that income elasticity is higher at low-income levels and lower at higher income levels. Other recent studies, including (Bloom, Canning, & Sevilla, 2004), (Erdil & Yetkiner, 2004), (Dreger & Reimers, 2005), (Sen, 2005), (Yu & Chu, 2007), (Chakroun, 2009), (Erdil & Yetkiner, 2009), (Wang, 2009), (Baltagi & Moscone, 2010), and (Hartwig, 2010) do not lend support to the view that health capital formation fosters long-term economic growth in the OECD. (Swift, 2011) used a Johansen multivariate cointegration analysis to examine the relationship between health and GDP for 13 OECD countries. (Narayan, Narayan, & Smyth, 2011) examined whether real health expenditure is a luxury for (OECD) countries over the 1972 to 2004 within a panel unit root and panel cointegration. (Farag et al., 2012) used a panel data set for 173 countries for the 1995-2006 period and found that health care has an income elasticity that qualifies it as a necessity. (Fuchs, 2013) concluded that the rate of growth in health care expenditures appears to have been substantially related to the growth of the GDP. (Lorenzoni, Belloni, & Sassi, 2014), (Rtveladze et al., 2014), (Ozturk & Topcu, 2014), (Lv & Zhu, 2014), and (Pascual & Gonzaacute, 2014) demonstrated the relationship between health expenditures and economic growth in OECD countries and other regions in the world. The findings of these articles indicate that GDP is the most important factor determining health care expenditures of countries. After a preliminary exploration of the data, we first assessed whether our variables were non-stationary; next, we tested whether our variables formed a cointegrating set and therefore, whether they are linked in the long-run.

### 1.1 Data Description

This article revisits the subject of existence unit roots in the OPEC data and long-run relationship between health spending and GDP in OPEC. We investigated the stationary and cointegration properties between health expenditures and GDP. We also examined the stationary and cointegration of logarithm health expenditures and logarithm of GDP based on the panel cointegration analysis (LHE is logarithm of health expenditures total per capita and LGDP is logarithm of GDP per capita) for a sample of 12 OPEC countries using data for the period 1995-2012 (t=18). We gathered data on per capita total health care expenditures and per capita income estimated in GDP. We also collected data from a world development indicator for public expenditures on health and private expenditures on health care in these countries the data (Group, 2012). There are very few paper concerning the relationship between health expenditures and GDP for non OECD countries (Mehrara & Fazaeli, 2009). We report some health and economic index for 12 countries listed in Table 1. Qatar has the most income (92801 US$ per capita) and the most spending on health care (2029 US$ per capita) among the studied countries. Nigeria, on the other hand, has the lowest income and health expenditures (2742 and 94 US$ per capita). OPEC average income is 22602 US$ per capita; that is, 60 % OECD average (37768 US$) but OPEC average health spending is 701 US$ per capita; that is, 20 % OECD average (3407). OPEC average public health expenditures is 6.84 % by government expenditures, whereas OECD average is 15.16. In addition, OPEC average out of pocket of total health spending is 28.1 % and OECD average is 16.49 %. Data shows that the share of private sector health expenditures is not large in OPEC countries because they have oil income.

**Table 1:** Health economic data in OPEC (2012)

Country	GDP per capita (current US$)	Health expenditure per capita (current US$)	Health expenditureprivate (% GDP)	Health expenditurepublic (% GDP)	Health expenditure public (%gov-exp)	Out of pocket (%total)
Algeria	5540	190	0.83	4.4	9.8	15.0
Angola	5310	279	1.31	2.2	5.6	26.7
Ecuador	5656	361	3.53	2.9	7.1	51.4
U.A Emirates	40444	1343	0.92	2.7	9.3	20.4
Iran	6578	490	4.00	1.9	15.4	52.5
Iraq	6632	226	1.67	1.9	4.4	46.4
Kuwait	53544	1428	0.44	2.1	5.6	15.8
Libya	13303	578	0.89	3.0	6.9	22.7
Nigeria	2742	94	4.18	1.9	6.7	65.9
Qatar	92801	2029	0.35	1.8	5.3	8.5
Saudi Arabia	25946	795	1.10	2.1	5.7	18.7
Venezuela, RB	12729	593	3.08	1.6	5.5	63.7
OPEC average	22602	701	1.37	2.27	6.84	28.1
OECD average	37768	3407	2.33	6.72	15.16	16.49

*Source:* WDI (2012).

## 2. Methodology

A series is said to be stationary if the mean and auto covariances of the series do not depend on time. Any data series that is has unit root is said to be non stationary. LHE and LGDP are time series so we have to test as stationary (M. Mehrara & Fazaeli, 2012). Dickey and Fuller (1979) demonstrated that in the hypothesis of a unit root, this statistic does not follow the conventional Student’s t-distribution. Panel unit root test is (ADF) individual unit root tests to a common panel unit root test (Banerjee, 1999). A difference stationary series is said to be integrated and is denoted as I (d) where d is the order of integration.


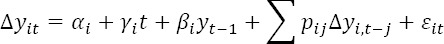


(Im, Pesaran, & Shin, 2003), (Maddala & Wu, 1999), (Choi, 2001), & (Hadri, 2000) are the other tests for unit root. Pedroni (2000, 2004) proposed several tests for the null hypothesis of no cointegration in a panel data model (Pedroni, 2004).

We applied a cointegration approach to panel data model with fixed coefficients in order to determine the relation between GDP and health expenditures per capita.


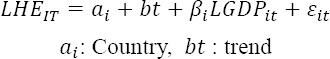


*a_i*: Country, *bt* : trend

ɛ_it_ = estimated residual indicating deviations from the long-run relationship.

To test the hypothesis of non cointegration we used Error Correction Model and estimated (Breitung, 2002):


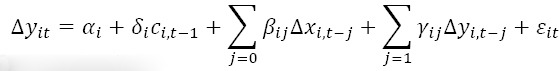


To estimate long-run & short-run elasticity:


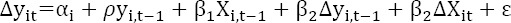


## 3. Results

In the first step, we examined unit roots in LHE and LGDP. In the next step we looked for a relationship between LGDP and LHE using the panel cointegration (Engle-Granger) test (Granger & Lin, 1995). Unit root and trend stationary results indicated that both LHE and LGDP are non-stationary (Table 2). The findings indicated that LHE and LGDP are co integrated (I(1)).The panel cointegration tests point to the existence of a long-run relationship.The coefficient of the LGDP is showed as an estimate of the income elasticity of health. If the income elasticity is between 0 and 1, that good is defined as a necessity, and if greater than 1, it is a luxury good. We obtain long-run elasticity is 0.89. This means health care is not a luxury good in these countries (Table 3).

**Table 2:** Unit root test

Unit root test for LHE	Unit root test for LGDP			
Test method	Test statistic	Result	Test statistic	Result
ADF - Fisher	16.17(0.88)	No stationary	15.08(0.77)	No stationary

**Table 3:** Panel cointegration tests

Test method	Test statistic (prob-value)	Result	Long-run (prob-value)
ADF	5.88 (0.0002)	Cointegration	0.89 (0.003)

## 4. Conclusion

This paper investigated the relationship between health spending and GDP per capita in the OPEC countries over 18 years. We studied the stationary and cointegration of health expenditures and GDP. We examine the stationary and cointegration of health expenditures and GDP, using stationary and cointegration tests. Our study demonstrates that health expenditures and GDP have unit root. Cointegration test results showed strong relationship between health expenditures and GDP. These results imply that health is not a luxury good in oil exporting countries. The common factor regressions indicate income elasticity below unity. In spite of high health expenditures per capita, in these countries, the share of the health sector from GDP is not very large.

## References

[ref1] Anderson G. F, Hurst J, Hussey P. S, Jee-Hughes M (2000). Health spending and outcomes: Trends in OECD countries, 1960-1998. Health Affairs.

[ref2] Baltagi B. H, Moscone F (2010). Health care expenditure and income in the OECD reconsidered: Evidence from panel data. Economic Modelling.

[ref3] Banerjee A (1999). Panel data unit roots and cointegration: An overview. Oxford Bulletin of economics and Statistics.

[ref4] Bloom D. E, Canning D, Sevilla J (2004). The effect of health on economic growth: A production function approach. World development.

[ref5] Breitung J (2002). Nonparametric tests for unit roots and cointegration. Journal of econometrics.

[ref6] Chakroun M (2009). Health care expenditure and GDP: An international panel smooth transition approach.

[ref7] Choi I (2001). Unit root tests for panel data. Journal of international money and Finance.

[ref8] Devlin N, Hansen P (2001). Health care spending and economic output: Granger causality. Applied Economics Letters.

[ref9] Di Matteo L (2003). The income elasticity ofhealth care spending. The European Journal of Health Economics.

[ref10] Dreger C, Reimers H.-E (2005). Health care expenditures in OECD countries: A panel unit root and cointegration analysis. IZA Discussion paper series.

[ref11] Erdil E, Yetkiner I. H (2004). A panel data approach for income-health causality. FNU-47.

[ref12] Erdil E, Yetkiner I. H (2009). The Granger-causality between health care expenditure and output: A panel data approach. Applied Economics.

[ref13] Farag M, NandaKumar A, Wallack S, Hodgkin D, Gaumer G, Erbil C (2012). The income elasticity of health care spending in developing and developed countries. International Journal of Health Care Finance and Economics.

[ref14] Fuchs V. R (2013). The gross domestic product and health care spending. New England Journal of Medicine.

[ref15] Gerdtham U.-G, Löthgren M (2002). New panel results on cointegration of international health expenditure and GDP. Applied Economics.

[ref16] Granger C. W, Lin J.-L (1995). Causality in the long run. Econometric theory.

[ref17] Group W. B (2012). World Development Indicators 2012: World Bank Publications.

[ref18] Hadri K (2000). Testing for stationarity in heterogeneous panel data. The Econometrics Journal.

[ref19] Hansen P, King A (1996). The determinants of health care expenditure: A cointegration approach. Journal of Health Economics.

[ref20] Hartwig J (2010). Is health capital formation good for long-term economic growth?-Panel Granger-causality evidence for OECD countries. Journal of macroeconomics.

[ref21] Im K. S, Pesaran M. H, Shin Y (2003). Testing for unit roots in heterogeneous panels. Journal of econometrics.

[ref22] Lorenzoni L, Belloni A, Sassi F (2014). Health-care expenditure and health policy in the USA versus other high-spending OECD countries. The Lancet.

[ref23] Lv Z, Zhu H (2014). Health Care Expenditure and GDP in African Countries: Evidence from Semiparametric Estimation with Panel Data. The Scientific World Journal.

[ref24] Maddala G. S, Wu S (1999). A comparative study of unit root tests with panel data and a new simple test. Oxford Bulletin of economics and Statistics.

[ref25] McCoskey S. K, Selden T. M (1998). Health care expenditures and GDP: Panel data unit root test results. Journal of Health Economics.

[ref26] Mehrara, Fazaeli A (2009). Relationship between health expenditures and economic growth in MENA countries. Faslname-Modiraite Salamat.

[ref27] Mehrara M, Fazaeli A. A (2012). The Relationship between Health Expenditures and Economic Growth in Middle East & North Africa (MENA) Countries. International Journal of Business Management & Economic Research.

[ref28] Narayan P. K, Narayan S, Smyth R (2011). Is health care really a luxury in OECD countries?Evidence from alternative price deflators. Applied Economics.

[ref29] Newhouse J. P (1977). Medical-care expenditure: a cross-national survey. Journal of human resources.

[ref30] Ozturk S, Topcu E (2014). Health Expenditures and Economic Growth: Evidence from G8.

[ref31] Parkin D, McGuire A, Yule B (1987). Aggregate health care expenditures and national income: Is health care a luxury good?. Journal of health economics.

[ref32] Pascual M, Gonzaacute N (2014). About the relationship between health expenditure and GDP: More evidence. African Journal of Business Management.

[ref33] Pedroni P (2004). Panel cointegration: Asymptotic and finite sample properties of pooled time series tests with an application to the PPP hypothesis. Econometric theory.

[ref34] Rtveladze K, Marsh T, Barquera S, Sanchez Romero L. M, Levy D, Melendez G, Brown M (2014). Obesity prevalence in Mexico: Impact on health and economic burden. Public health nutrition.

[ref35] Sen A (2005). Is health care a luxury? New evidence from OECD data. International Journal of Health Care Finance and Economics.

[ref36] Swift R (2011). The relationship between health and GDP in OECD countries in the very long run. Health economics.

[ref37] Wang Z (2009). The determinants of health expenditures: Evidence from US state-level data. Applied Economics.

[ref38] Yu T. H.-K, Chu H. Y (2007). Is health care really a luxury? A demand and supply approach. Applied Economics.

